# Efficient Massive Computing for Deformable Volume Data Using Revised Parallel Resampling

**DOI:** 10.3390/s22166276

**Published:** 2022-08-20

**Authors:** Chailim Park, Heewon Kye

**Affiliations:** Division of Computer Engineering, Hansung University, Seoul 02876, Korea

**Keywords:** massive computing for volume deformation, parallel resampling, GPU parallel computing, low-latency image generation, IoE medical simulation

## Abstract

In this paper, we propose an improved parallel resampling technique. Parallel resampling is a deformable object generation method based on volume data applied to medical simulations. Existing parallel resampling is not suitable for massive computing, because the number of samplings is high and floating-point precision problems may occur. This study addresses these problems to obtain improved user latency when performing medical simulations. Specifically, instead of interpolating values after volume sampling, the efficiency is improved by performing volume sampling after coordinate interpolation. Next, the floating-point error in the calculation of the sampling position is described, and the advantage of barycentric interpolation using a reference point is discussed. The experimental results showed a significant improvement over the existing method. Volume data comprising more than 600 images used in clinical practice were deformed and rendered at interactive speed. In an Internet of Everything environment, medical imaging systems are an important application, and simulation image generation is also valuable in the overall system. Through the proposed method, the performance of the whole system can be improved.

## 1. Introduction

Virtual medical procedures are being applied to clinical education and surgical planning, contributing to the improvement in medical services on the Internet of Everything (IoE). Generally, virtual medical procedures concern performing simulations of deforming human body data using volume-based and surface-based methods for volume deformation and rendering. Surface-based methods are advantageous in that they require fewer computational data and are faster compared to other methods. This is because the computation considers only the surface of the object. However, it has a disadvantage in that it is difficult to apply to topological changes such as cutting or merging. Although cutting has been studied extensively [1,2,3], merging is a difficult problem.

Medical simulation involves operations such as incision and suturing and, thus, it is appropriate to apply a volume-based deformation method that is independent of topology changes. Due to the large size of volume data, calculating the deformation is time consuming, and more efficient methods are desired. Recently, parallel deformation algorithms using GPU (chainmail [4], mass-spring [5], position-based dynamics [6], etc.) have been well used.

It is important to visualize the deformed data along with the deformation calculation. To render more precise results, the volume resolution needs to be expressed as large as 512^3^ and, thus, visualization becomes time consuming. In this study, we visualized high-resolution deformed volumes by utilizing GPU parallelization.

The visualization of volume deformation is largely divided into two types (assuming that the general ray-casting method is used). The first method generates deformed viewing rays while leaving the volume data unchanged, and the second method generates new deformed volume data using straight viewing rays.

The first method performs iterative sampling for each ray in the original data. To calculate each sampling position in a deformed viewing ray, the inverse transform of the user deformation needs to be calculated, which becomes difficult when calculating a large number of inverse transforms. It becomes difficult to handle exceptions, such as the cut region, where inverse transformations do not exist, and the process is slow and potentially erroneous due to the use of iterative numerical solutions such as the Newton–Raphson or gradient-descent-like methods [7].

This issue has been addressed in the literature. When only a portion of the entire volume data was deformed, the screen area corresponding to the deformed portion was calculated in advance, and supersampling was applied to that area during rendering in [8]. In [9], an efficient approach to decompose the entire volume unequally was proposed. However, since the inverse transformation vector was simply defined as the opposite vector of the forward transformation, a large error may occur when the amount of change in the transformation vector is large. Since the inverse transformation does not exist in the cut region, this exception can be handled by making a special mark in the region where the inverse transformation does not exist. In [10], this problem was solved by introducing an alpha volume indicating the cut region. In [11], it was considered that when sampling was performed through inverse transform, and errors occur in the interpolation and gradient calculation process. In their study, image quality improvement in fine areas was realized by interpolation using a nonlinear higher-order function and by considering deformation in the gradient calculation.

The second method is to directly create deformed volume data from original volume data. If point-based forward mapping is applied, holes or overlapping problems generally occur. Although image-based backward mapping is a possible solution [12], it is time consuming to identify corresponding particles for each output grid position. Therefore, we used tetrahedron-based forward mapping using rasterization to address this problem. This technique was not considered feasible previously due to the high computation times, but recent advances in GPUs have made it possible. Parallel resampling is a typical tetrahedron-based forward mapping method for volume deformation using GPU parallelization.

For reference, the term parallel resampling is also used for particle filter techniques [13,14], which are common methods used to estimate the evolving state of nonlinear, non-Gaussian time-variant systems. However, the parallel resampling used in this study was different from the above studies, as it is a volume-based sampling technique. The basic parallel resampling method [15,16] cannot handle many tetrahedra due to the fact of its performance limitations. To implement more sophisticated deformations, new methods should be explored. In this study, we generated deformed volume data at a high speed by considering parallel resampling. By improving the resolution of the deformation, 512^3^ data were deformed and visualized in real time.

The contributions of this paper are as follows:

1. We propose an efficient volume deformation computing for massive data;
2. User latency was improved through a high-speed deformable object creation algorithm;
3. We present a more reliable barycentric interpolation method suitable for GPUs.

### Overall Flow of Our System

**Figure 1:**
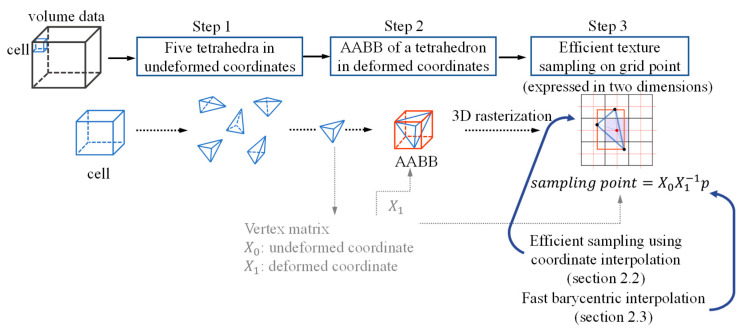
Overall flow of the proposed method.

The overall flow of this study is illustrated in three steps as shown in Figure 1. (Step 1) The entire volume data that needed to be transformed were composed of cells that were hexahedrons of a fixed size. Each cell was decomposed into five tetrahedra. The vertex matrices, *X*_0_ and *X*_1_, were created using the coordinate values of the four vertices constituting one tetrahedron as column vectors. For reference, it was assumed that the coordinate values after deformation corresponding to *X*_1_ were already calculated through simulation methods such as 3D chainmail [4] or mass-spring [5], and physical simulation was not within the scope of this study. (Step 2) The goal of this study was to store the resampling value for each grid point inside every tetrahedron to generate the entire deformed volume data. Therefore, first, in order to quickly extract the area inside the tetrahedron, the axis-aligned bounding box (AABB) of the tetrahedron was calculated in deformed coordinates (Step 3). For each grid point belonging to the AABB region, it was tested whether the grid point was inside the tetrahedron. The resampling value was calculated at the grid points that passed the test.

The structure of this paper is as follows: Section 2.1 describes Steps 1 and 2 of Figure 1 as a related study. In this study, Step 3 of Figure 1 is efficiently performed by applying the two proposed methods. Section 2.2 describes how to efficiently calculate resampling values, and Section 2.3 describes how to efficiently calculate the resampling position using the coordinate system. Next, in Section 3, the experimental results are presented, and in Section 4, the conclusions are drawn.

## 2. Materials and Methods

### 2.1. Related Work—Parallel Resampling

**Figure 2:**
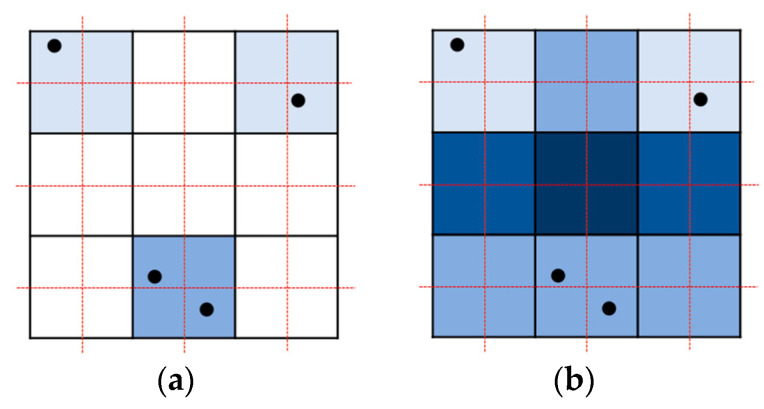
Forward mapping with problems: (**a**) holes and overlapping in a point-based manner; (**b**) messy overlapping in the splatting method.

Parallel resampling is a method of storing forward mapping results in new volume data. When forward mapping is performed in a point-based manner, as shown in Figure 2a, problems such as overlaps or holes occur. If a kernel filter is used instead of a point, as shown in Figure 2b, overlap occurs a different number of times for each pixel, and parallelization becomes difficult.

**Figure 3:**
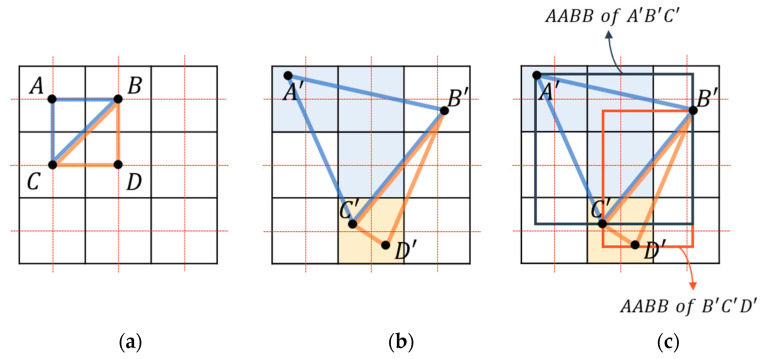
Rasterization process: (**a**) triangles comprising grid points in undeformed space; (**b**) transformed into deformed space for resampling when the grid point is included in the triangle; (**c**) judgment performed on the grid points within the axis-aligned bounding box (AABB) of each triangle.

If rasterization is performed by connecting these points to a triangle, holes and overlaps can be avoided, and parallelization is also possible. The four vertices constituting the rectangle in the undeformed space are transformed into two adjacent triangles in the deformed space (Figure 3a,b). Since the output occurs only when the center of the pixel in the deformed space is in the triangle, the resampling operation occurs only once at each output coordinate.

**Figure 4:**
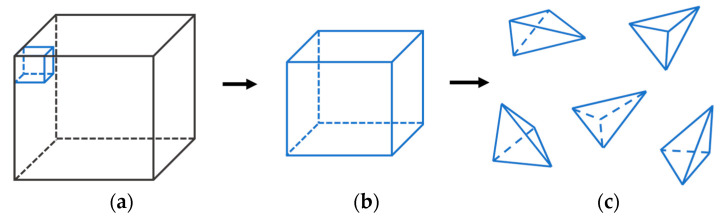
(**a**) Volume data; (**b**) a group of cells; (**c**) each cell is decomposed into 5 tetrahedra.

In three dimensions, eight vertices constitute a hexahedral cell. As shown in Figure 4, the cell is divided (Figure 4a,b) into 5 tetrahedra (Figure 4c).

Each tetrahedron is transformed by changing the coordinates of each vertex comprising the cell. The process of resampling inside each tetrahedron is as follows. For example, it is assumed that 2^3^ voxels constitute one cell. Volume data with a size of L × M × N voxels comprise (L – 1) × (M – 1) × (N – 1) cells. If the cell comprises B^3^ voxels, the volume data comprise approximately L/B × M/B × N/B cells. A cell is decomposed into five tetrahedra regardless of the cell size, and the size of each tetrahedron is proportional to the cell size.

Whether the output voxels are inside the transformed tetrahedron is determined using the barycentric coordinates, and resampling is performed at each voxel position inside the tetrahedron. The area near the tetrahedron is defined by the AABB of the tetrahedron, as shown in Figure 3c. For each candidate voxel inside the AABB, the barycentric coordinates (for the four vertices of the tetrahedron) are calculated. Since the calculated value (***b***) is the barycentric coordinates in the three-dimensional space, it is expressed as a four-dimensional vector. When each component of the vector is between 0 and 1, it is determined to be inside a tetrahedron.

Representative existing studies using this approach include [15,16]. A tetrahedron was generated using a relatively large cell in [15], and a cell with the same voxel size was generated in [16]. This parallel resampling method can be performed in real time using a touch screen [17], and it can be applied by generating a tetrahedral mesh in an intermediate step [18] when generating volume data from a general mesh.

### 2.2. Efficient Sampling Using Coordinate Interpolation

In this study, we aimed to improve the sampling performance by combining the advantages of parallel resampling, Gascon’s method [15], and Aguilera’s method [16]. Moreover, the characteristics of the two previous studies are explained, and the differences from this study are shown. For convenience, each thread of the GPU is expressed as a thread, and the combined bundle of threads is expressed as a thread block.

Gascon’s method assumes that the size of each tetrahedron is suitably large (more than a few tens of voxels in size). Therefore, one or several thread blocks correspond to each tetrahedron. Threads belonging to one thread block can share the information of the tetrahedron ($\bar{X_{0}}$, bounding box). The transformation of the tetrahedron is performed in the CPU, because the total number of tetrahedra was fewer than 5000 in Gascon’s study. However, the number of tetrahedra has to be significantly increased in order to achieve smooth movement of deformation. In this study, one tetrahedron was assumed to be as small as the voxel size and, thus, there was no reason to configure one tetrahedron as a thread block and activate hundreds of threads.

Aguilera’s method defines a vertex as a coordinate in deformed space and a density value. Coordinates in deformed space use the precalculated simulation results. A cell is a hexahedron with eight voxels as vertices, and the eight density values are obtained by performing texture sampling at each voxel position. Resampling concerns interpolating the density values stored at vertex positions. The resampling value at the grid points in the transformed space is calculated and stored in the deformed volume data. Aguilera’s method [16] is different from Gascon’s method [15]. In Gascon’s method, one large tetrahedron contains several cells, whereas in Aguilera’s method, one cell is decomposed into five very small tetrahedra. Each thread is used to process one cell, i.e., five tetrahedra.

In our study, we constructed a high-speed algorithm to generate precise results by combining only the advantages of the two previous studies. Aguilera’s method was used for each thread processing one cell, which was decomposed into five small tetrahedra. Gascon’s method was used for texture sampling in the rasterization step instead of sampling eight times for each cell in the modeling step. Our method is efficient because the tetrahedron is small, and the actual resampling in a small tetrahedron is infrequent.

**Figure 5:**
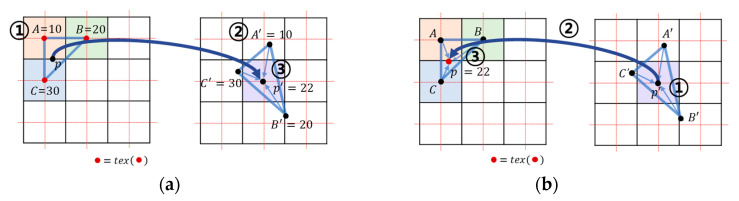
Texture sampling method: (**a**) Aguila’s method performs (1) sampling (2) interpolating and (3) writing; (**b**) the proposed and Gascon’s method performs (1) interpolating (2) sampling and (3) writing .

Each thread is in charge of one cell to perform parallel processing. One cell is decomposed into five tetrahedra, and calculation is performed for each tetrahedron. The resampling is calculated at every grid point inside the AABB of each tetrahedron in deformed space (output volume data). The coordinates are obtained by the weighted average of the four tetrahedron vertices. Aguilera’s method calculates the weighted average of the brightness values (Figure 5a ③), while Gascon’s method calculates the weighted average of the coordinates in the undeformed space (Figure 5b ②). In this study, texture sampling was performed at the interpolated coordinates according to Gascon’s method (Figure 5b ③). This method reduces the number of texture sampling compared to Aguilera’s method. Since the cell size is 1 in undeformed space, on average, texture writing will occur only once for each cell, although one cell comprises five tetrahedrons.

As many threads as the number of hexahedral cells are launched, if we assume that the size of volume data is (*volx*, *voly*, and *volz*):

number of threads = number of cells = (*volx* – 1)∙(*voly* – 1)∙(*volz* – 1) (1)

Note that five tetrahedra are created for each cell:

number of tetrahedra = 5∙(*volx* – 1)∙(*voly* – 1)∙(*volz* – 1) (2)

The number of output voxels is *volx*∙*voly*∙*volz* with the same size as the input voxel. Since the tetrahedra are adjacent to each other without overlapping, the maximum number of resampling and writing occurs in *volx*∙*voly*∙*volz*, which is the size of the output data. The number of outputs for one cell is approximately 1. Compared to Aguilera’s method, where texture sampling occurs eight times for one cell, the proposed method is more efficient.

(3) $$\frac{\left(volx\right)\cdot \left(voly\right)\cdot \left(volz\right)}{\left(volx-1\right)\cdot \left(voly-1\right)\cdot \left(volz-1\right)}\approx 1$$

We store both positions before and after deformation for the eight vertices of the cell, because resampling is performed after the deformation. The data required for each thread are 8 (vertices per cell) × 2 (before and after movement) × 3 (x, y, z) × 4 (size of float) = 192 bytes. In Aguilera’s method, it is 8 (vertices per cell) × (3 (x, y, z) × 4 (size of float) + 2 (size of density value)) = 112 bytes. The proposed method uses slightly more memory than the existing method. For reference, Gascon’s method shares the coordinates of a tetrahedron before deformation for each block; thus, the coordinates before deformation can be read from a precalculated memory. Gascon’s method seems to require less memory, but it can be used only when the number of tetrahedra is small.

The last step of generating deformation data is to perform sampling and store each sampling value in the target volume data. Equation (4) is a matrix comprising the coordinates of the four vertices of a tetrahedron, where $\bar{X_{0}}$ is generated with coordinates in undeformed space, and $\bar{X_{1}}$ is generated with coordinates in deformed space. Sampling is performed at the coordinates, *x*_0_, before deformation, which is obtained from the coordinates *x*_1_ of the grid point after deformation. Since the transformation of one tetrahedron is assumed to be an affine transform, the barycentric coordinates of *x*_0_ and the barycentric coordinates of *x*_1_ are the same as in Equations (5) and (6).

(4) $$\begin{array}{c} X_{0}=\left(\begin{array}{cccc} A & B & C & D \end{array}\right)=\left(\begin{array}{cc} \begin{array}{cc} A_{x} & B_{x}\\ A_{y} & B_{y}\\ A_{z} & B_{z} \end{array} & \begin{array}{cc} C_{x} & D_{x}\\ C_{y} & D_{y}\\ C_{z} & D_{z} \end{array} \end{array}\right)\\ \bar{X_{0}}=\left(\begin{array}{c} X_{0}\\ 1^{T} \end{array}\right)=\left(\begin{array}{cccc} A_{x} & B_{x} & C_{x} & D_{x}\;\\ A_{y} & B_{y} & C_{y} & D_{y}\\ A_{z} & B_{z} & C_{z} & D_{z}\\ 1 & 1 & 1 & 1 \end{array}\right)\;\\ X_{1}=\left(\begin{array}{cc} \begin{array}{cc} A^{\prime } & B^{\prime } \end{array} & \begin{array}{cc} C^{\prime } & D^{\prime } \end{array} \end{array}\right)=\left(\begin{array}{cc} \begin{array}{cc} {A^{\prime }}_{x} & {B^{\prime }}_{x}\\ {A^{\prime }}_{y} & {B^{\prime }}_{y}\\ {A^{\prime }}_{z} & {B^{\prime }}_{z} \end{array} & \begin{array}{cc} {C^{\prime }}_{x} & {D^{\prime }}_{x}\\ {C^{\prime }}_{y} & {D^{\prime }}_{y}\\ {C^{\prime }}_{z} & {D^{\prime }}_{z} \end{array} \end{array}\right)\\ \bar{X_{1}}=\left(\begin{array}{c} X_{1}\\ 1^{T} \end{array}\right)=\left(\begin{array}{cccc} {A^{\prime }}_{x} & {B^{\prime }}_{x} & {C^{\prime }}_{x} & {D^{\prime }}_{x}\;\\ {A^{\prime }}_{y} & {B^{\prime }}_{y} & {C^{\prime }}_{y} & {D^{\prime }}_{y}\\ {A^{\prime }}_{z} & {B^{\prime }}_{z} & {C^{\prime }}_{z} & {D^{\prime }}_{z}\\ 1 & 1 & 1 & 1 \end{array}\right) \end{array}$$

(5) $$\left(\begin{array}{c} x_{0}\\ 1 \end{array}\right)=\bar{X_{0}}\cdot b,\;\;b={\bar{X_{0}}}^{-1}\left(\begin{array}{c} x_{0}\\ 1 \end{array}\right)$$

(6) $$\left(\begin{array}{c} x_{1}\\ 1 \end{array}\right)=\bar{X_{1}}\cdot b,\;\;b={\bar{X_{1}}}^{-1}\left(\begin{array}{c} x_{1}\\ 1 \end{array}\right)$$

(7) $$\left(\begin{array}{c} x_{0}\\ 1 \end{array}\right)=\bar{X_{0}}\cdot b=\bar{X_{0}}\cdot {\bar{X_{1}}}^{-1}\left(\begin{array}{c} x_{1}\\ 1 \end{array}\right)$$

To obtain the barycentric coordinates, the coordinates *x_1_* of the voxel (Equation (6)) are defined in the form of a four-dimensional homogeneous coordinate and multiplied by the inverse of the matrix comprising four column vectors of the tetrahedral vertex coordinates. As shown in Equation (7), *x*_0_ is obtained by multiplying the barycentric coordinates ***b*** by ***X*_0_**, which is the column vector matrix using four vertices of a tetrahedron in undeformed coordinates. The output data are generated with the value obtained by texture sampling on the undeformed coordinates.

This can be expressed as an algorithm (Algorithms 1 and 2) as follows:


**Algorithm 1** Parallel Resampling of Aguilera’s Method [16]  1:  **struct vertex**
  2:  **float** x,y,z;  3:  **short** value; /* value has already been resampled */  4:  **procedure** SampleTetrahedron (**vertex**
***A***, ***B***, ***C***, ***D***, Tex3D outGrid)  5:   **aabb** boundingBox = outGrid.computeAABB(***A***, ***B***, ***C***, ***D***);  6:   **foreach** (voxel **in** boundingBox)  7:    **float4** baryCoords = computeBaryCoords (voxel.center, ***A***, ***B***, ***C***, ***D***);  8:    **if** (centerLiesInsideTetrahedron (baryCoords))  9:     **short** newValue = interpolateValue (baryCoords, ***A***, ***B***, ***C***, ***D***); 10:     setValue (voxel, newValue); 11:    **end if** 12:   **end foreach** 13: **end procedure**
**Algorithm 2** Parallel Resampling of Proposed Method  1:  **struct vertex**
  2:  **float** x,y,z;   3:  **float** tx,tx,tz; /* original position */  4:  **procedure** SampleTetrahedron (**Mat4** $\bar{X_{0}}$, **vertex**
***A***, ***B***, ***C***, ***D***, Tex3D outGrid, Tex3D inVolume)  5:   **aabb** boundingBox = outGrid.computeAABB(***A***, ***B***, ***C***, ***D***);  6:   **foreach** (voxel **in** boundingBox)  7:    **float4** baryCoords = computeBaryCoords (voxel.center, ***A***, ***B***, ***C***, ***D***);  /* $b={\bar{X_{1}}}^{-1}\left(\begin{array}{c} x_{1}\\ 1 \end{array}\right)$ in Equation (3) */  8:    **if** (centerLiesInsideTetrahedron(baryCoords))  9:     **float4** inpos = $\bar{X_{0}}$ * baryCoords; 10:     **float4** newValue = tex3D (inVolume, inpos.xyz); 11:    setValue (voxel, newValue); 12:   **end if** 13:  **end foreach** 14: **end procedure**


### 2.3. Efficient Barycentric Interpolation for a Massive Number of Tetrahedra

As described in Equation (7), the calculation of the inverse matrix occurs for each tetrahedron. Since we considered the large number of 786 M (= 512^2^ × 600 × 5) tetrahedra (Aguilera used 65 M tetrahedra [16]), efficient computation is required. Here, we explain the importance of efficient inverse matrix computation and discuss the numerical instability that occurs when the number of tetrahedra increases.

#### 2.3.1. Barycentric Interpolation and Inverse Matrix

In this study, the coordinates in undeformed space were calculated using barycentric coordinates, and sampling was performed for each output grid point. As expressed by *computeBaryCoords* in Algorithm 1, the barycentric coordinates are calculated using the inverse matrix (Equation (6)). It is necessary to calculate the inverse matrix for each tetrahedron, but as the number of tetrahedra increases and the size decreases, it becomes numerically unstable. In the following example, the coordinates of the four points constituting a tetrahedron are ***A*** (255.9, 256.7, and 133.1), ***B*** (256.7, 255.9, and 133.4), ***C*** (256.7, 256.7, and 132.3), and ***D*** (255.9, 255.9, and 132.3). Using each point as a column vector, the inverse of the matrix $\bar{X_{1}}$ is obtained as:

(8) $$\bar{X_{1}}=\left[\begin{array}{cc} \begin{array}{cc} 255.9 & 256.7\\ 256.7 & 255.9 \end{array} & \begin{array}{cc} 256.7 & 255.9\\ 256.7 & 255.9 \end{array}\\ \begin{array}{cc} 133.1 & 133.4\\ 1 & 1 \end{array} & \begin{array}{cc} 132.3 & 132.3\\ 1 & 1 \end{array} \end{array}\right]$$

However, if the inverse matrix of $\bar{X_{1}}$ is calculated with a single-precision floating point (float), an error occurs. Appendix A shows finding the determinant, which is the first step to finding the inverse matrix. The correct determinant value is 1.216, but the calculation value using float is 2.010187, which shows an obvious error. The reason for this is that the formulas in the form of *a·b·c–d·e·f* are repeated to calculate the inverse matrix. Both *a·b·c* and *d·e·f*, which are the result of multiplying the coordinate values, are respectively large values (>10^6^). However, since the result of *a·b·c–d·e·f* is small (<10), an error easily occurs when using float. In our study, since each cell was small, the coordinate values of the adjacent vertices constituting a tetrahedron were similar. As the number of cells is increased, this error becomes more prominent, and the result becomes unusable.

(9) $${\bar{X_{1}}}^{-1}=\left[\begin{array}{cc} \begin{array}{cc} -0.723684 & 0.526316\\ 0.723684 & -0.526316 \end{array} & \begin{array}{cc} 0.723684 & -0.526316\\ 0.526316 & -0.723684 \end{array}\\ \begin{array}{cc} 0.526316 & 0.526316\\ -69.631579 & -69.631579 \end{array} & \begin{array}{cc} -0.526316 & -0.526316\\ -250.243421 & 390.506579 \end{array} \end{array}\right]\;\mathrm{in}\;\mathrm{double}-\mathrm{precision}$$

(10) $${\bar{X_{1}}}^{-1}=\left[\begin{array}{cc} \begin{array}{cc} -0.437228\; & 0.318691\;\\ \;0.437228\; & 0.318691\; \end{array} & \begin{array}{cc} 0.437228 & -0.318691\\ \;0.318691 & -0.437228\; \end{array}\\ \begin{array}{cc} 0.322577 & 0.318691\;\\ -42.284821\; & -42.284821\; \end{array} & \begin{array}{cc} -0.322577 & -0.320634\\ -151.230408\; & 236.297531 \end{array} \end{array}\right]\;\mathrm{in}\;\sin\mathrm{gle}-\mathrm{precision}$$

The basic approach is to use the double-precision floating point (double). However, the double operation is significantly slower than the float operation, because a typical GPU contains less double-precision computing hardware. To solve this problem, we used a reference point for the barycentric coordinates described in next section.

#### 2.3.2. Calculation of the Barycentric Coordinates Using the Reference Point

**Figure 6:**
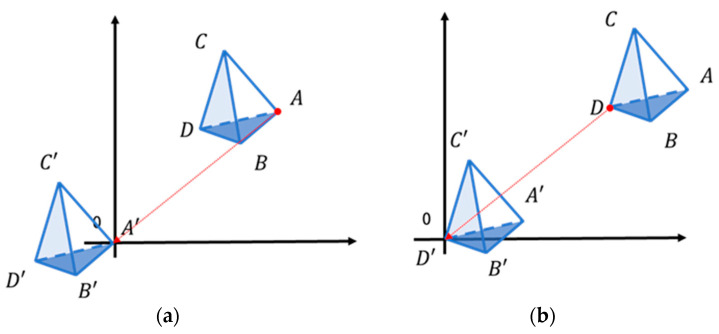
(**a**) Parallel movement of each point to the origin; (**b**) parallel movement of the last vertex to the origin.

In this study, the inverse matrix calculation was used only to obtain the barycentric coordinates, ***b***, to determine whether each point was inside the tetrahedron. Even when translating every point of the tetrahedron, the barycentric coordinates do not change. To keep the coordinate values as small as possible, we translated each point so that it was close to the origin (Figure 6a).

For convenience, the last vertex *D* among the four vertices of the tetrahedron was translated to the origin (Figure 6b), i.e., we calculated the barycentric coordinates with respect to D [19]. Since the size of the tetrahedron was very small, the coordinates of all points inside the AABB of the tetrahedron were located very close to the origin. Each value of *a·b·c* and *d·e·f* becomes smaller, and the error is negligible when float is used.

Equation (5) can be rewritten as follows:

(11) $$\left(\begin{array}{cccc} A_{x} & B_{x} & C_{x} & D_{x}\\ A_{y} & B_{y} & C_{y} & D_{y}\\ A_{z} & B_{z} & C_{z} & D_{z}\\ 1 & 1 & 1 & 1 \end{array}\right)\left(\begin{array}{c} b_{A}\\ b_{B}\\ b_{C}\\ b_{D} \end{array}\right)=\left(\begin{array}{c} X_{x}\\ X_{y}\\ X_{z}\\ 1 \end{array}\right)$$

Now, if all of the points, ***A***, ***B***, ***C***, ***D***, and ***X***, are moved in parallel by ***-D***, it can be expressed as:

(12) $$\begin{array}{ll} & \left(\begin{array}{cccc} A_{x}-D_{x} & B_{x}-D_{x} & C_{x}-D_{x} & D_{x}-D_{x}\\ A_{y}-D_{y} & B_{y}-D_{y} & C_{y}-D_{y} & D_{y}-D_{y}\\ A_{z}-D_{z} & B_{z}-D_{z} & C_{z}-D_{z} & D_{z}-D_{z}\\ 1 & 1 & 1 & 1 \end{array}\right)\left(\begin{array}{c} b_{A}\\ b_{B}\\ b_{C}\\ b_{D} \end{array}\right)\\ = & \left(\begin{array}{cccc} A_{x}-D_{x} & B_{x}-D_{x} & C_{x}-D_{x} & 0\\ A_{y}-D_{y} & B_{y}-D_{y} & C_{y}-D_{y} & 0\\ A_{z}-D_{z} & B_{z}-D_{z} & C_{z}-D_{z} & 0\\ 1 & 1 & 1 & 1 \end{array}\right)\left(\begin{array}{c} b_{A}\\ b_{B}\\ b_{C}\\ b_{D} \end{array}\right)=\left(\begin{array}{c} X_{x}-D_{x}\\ X_{y}-D_{y}\\ X_{z}-D_{z}\\ 1 \end{array}\right) \end{array}$$

Therefore, if only the 3 × 3 submatrix is observed, the following is obtained:

(13) $$\left(\begin{array}{ccc} A_{x}-D_{x} & B_{x}-D_{x} & C_{x}-D_{x}\\ A_{y}-D_{y} & B_{y}-D_{y} & C_{y}-D_{y}\\ A_{z}-D_{z} & B_{z}-D_{z} & C_{z}-D_{z} \end{array}\right)\left(\begin{array}{c} b_{A}\\ b_{B}\\ b_{C} \end{array}\right)=\left(\begin{array}{c} X_{x}-D_{x}\\ X_{y}-D_{y}\\ X_{z}-D_{z} \end{array}\right)$$

Here, ***b***^3^ (*b_A_*, *b_B_*, and *b_C_*) can be obtained by calculating only the inverse of the 3 × 3 matrix of (Equation (13)) instead of the 4 × 4 matrix. Moreover, the *b_D_* value is calculated using *b_A_ + b_B_ + b_C_ + b_D_* = 1. In the process of matrix inversion, the form *a·b–c·d* is used instead of *a·b·c—d·e·f*; therefore, we can use float without errors. Although calculation of the barycentric coordinates using the reference point is not a new proposal, it is worth highlighting that float can be used instead of double.

## 3. Results

### 3.1. Experimental Setup

The program was developed using C++ based on Visual Studio. Rendering was performed using ray casting [20] and parallelized with CUDA [21] on a laptop equipped with a GeForce GTX 1650 Mobile and a desktop computer equipped with a GeForce RTX 2080. The volume data used in the experiment were anonymized medical image CT data, and the details are shown in Table 1.

In this study, the transformation of the volume data is not of concern. We assumed that the transformation results existed and paid attention to generating volume data by parallel resampling. Therefore, we did not perform physics-based deformation separately but transformed the data with simple precalculated formulas. The three transformations generated for testing are as follows.

**Figure 7:**
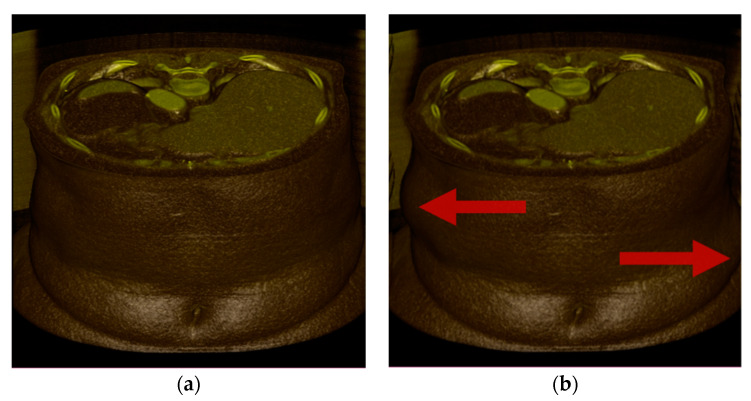

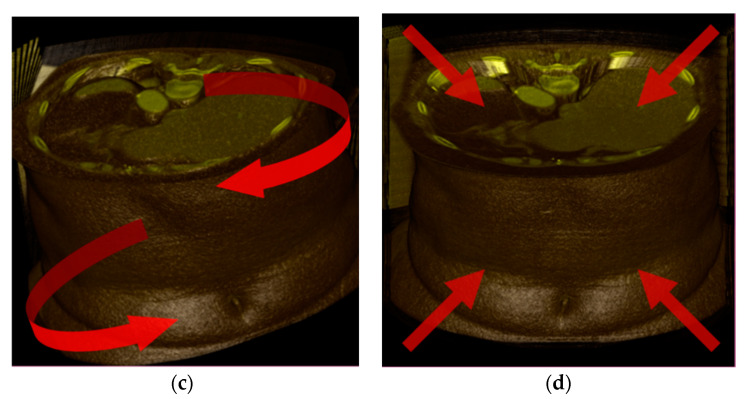
Test deformations: (**a**) undeformed; (**b**) Wave; (**c**) Twist; (**d**) Bubble. It is transformed in the direction of the red arrow.

The *Wave* transform (Figure 7b) performs transverse translation in the x-axis direction. The *Twist* transform ((Figure 7c) twists and rotates about the z-direction axis. The *Bubble* transform (Figure 7d) is expressed in the form of a sphere, and regional expansion and contraction occur. Assuming that the undeformed position is ***p***, the center point of the volume data is ***c***, the current time is *t*, and the deformed position is ***p′***, and each transformation is expressed as follows. In addition, ***x***, ***y***, and ***z*** are unit vectors in each axis direction.

(14) $$\mathrm{Wave}:\;p^{\prime }=p+\sin\left(p\cdot z+t\right)\cdot x$$

(15) $$\mathrm{Twist}:\;p^{\prime }=\left(\begin{array}{ccc} \cos\;\theta & -\sin\;\theta & 0\\ \sin\;\theta & \cos\;\theta & 0\\ 0 & 0 & 1 \end{array}\right)\left(p-c\right)+c\;\theta =\sin t\cdot \left(p-c\right)\cdot z$$

(16) $$\mathrm{Bubble}:\;p^{\prime }=\left(1+\frac{\sin t}{\left|p-c\right|}\right)\cdot \left(p-c\right)+c$$

Here, we show the effect of the proposed method using various testing transformations. In order to effectively reveal the experimental results, the experimental sequence was performed in the reverse order of the main sections. First, the results of the efficient barycentric interpolation method, described in Section 2.3, are presented, and then the effect of the sampling method proposed in Section 2.2 is shown.

### 3.2. Efficient Barycentric Interpolation

In order to show the efficiency of the barycentric interpolation method, described in Section 2.3, various methods for the inverse matrix calculation were analyzed. The *abdomen* data used and the average time of running 500 frames were measured. In Table 2, only the resampling time is indicated, and the rendering time was measured separately. The average rendering time was measured to be less than 1 ms on the desktop computer, and approximately 14 ms on the laptop. This is fast enough to enable real-time visualization.

The execution time according to different inverse matrix calculations was measured, and as shown in the Table 2, the execution speed of test (a) was the slowest. This was because the inverse of a 4 × 4 matrix requires significant computation. Calculating the barycentric coordinates with respect to a vertex [19] reduces the matrix size 4 × 4 to 3 × 3. Experiment (b) calculated the inverse of a 3 × 3 matrix using the double and, thus, the amount of computation was reduced significantly. As a result, compared to experiment (a), the speed improved by more than double. As explained in Section 2.3 on the relationship between data types and error, in the case of the experiment (c) using the float operation, the speed was improved by 4 to 6 times compared to experiment (b). It was surprising that the performance of the float on the GPU was higher compared to the double. Comparing the execution times of the proposed method (c) with the brute-force method (a), the performance improved by 10 to 22 times.

Although the proposed method improved the inverse matrix calculation speed, a different pattern was observed in the degree of improvement for the desktop and laptop computers. It improved by 14 to 22 times on the desktop and by 10 to 16 times on the laptop. In general, the graphics memory of laptops has a lower performance compared to desktops due to the fact of energy and heat problems. Therefore, the memory read/write bottleneck is more severe in a laptop. In the case of experiment (a), most of the time was consumed in calculating the inverse matrix comprising arithmetic operations. Further, in the case of experiment (c), the memory operations, such as resampling, become important, because the inverse matrix calculation has been optimized and reduced. Therefore, the performance improvement of (c) was partially reduced in the notebook. It was expected that the effect of the proposed method will be greater in hardware with high memory speed.

In the case of Wave, it was slightly faster than Twist or Bubble in experiment (a), because the calculation of Wave (Equation (14)) is simpler than that of the other equations. When we added some complex instructions to the Wave, the speed of Wave became similar to that of Twist and Bubble. For reference, the total execution time included the predefined deformation calculation time, inverse matrix calculation time, and data generation time using texture sampling. 

In order to confirm that there were no errors in the output image of the proposed method, the output images using the existing method and the proposed method were compared. In the case of the output image using the proposed method and the existing method, the values of the image difference were exactly equal to 0. As described in Section 2.3, if the coordinate values are kept small by moving the tetrahedron to the origin, the inverse matrix can be calculated with sufficient precision, even when we use small cells and float operations.

### 3.3. Efficient Sampling Using Coordinate Interpolation

The performance improvement was analyzed by applying the sampling method proposed in Section 2.2. The 3 × 3 float matrix calculation method, which showed the best performance in Table 2 column (c), was commonly applied to generate Table 3. The average time was measured for 500 frames. Aguilera’s method (a) obtained a weighted average from the eight sampled values for a cell, but the proposed method (b) sampled only once per output voxel by weighted averaging the coordinates in the original volume. When the proposed method was executed, it can be seen that the speed improved by 10–20% depending on the transform. Although the performance improvement was not significant, it was meaningful in that it provided additional performance improvement to the already optimized operation.

In the above experiment, the degree of performance improvement varied according to the transform. In the case of Bubble, since only a part of the data was transformed, the data reusability of the nonmoving part was high. Moreover, the Twist was complicated, as shown in Table 2. In addition, when multiple threads stored the deformed points using the Twist, the memory addresses to be stored were not contiguous due to the rotation, thereby reducing the locality and efficiency of memory access. In summary, the proposed method was more effective with local range deformation. In medical simulations, deformation occurs locally such as pulling or incising a part of human body data. The proposed method is more suitable for general medical surgery simulation.

**Figure 8:**
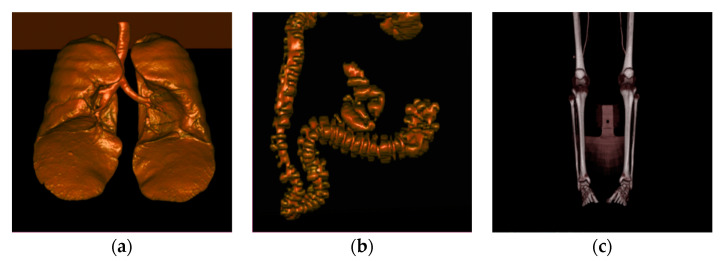
(**a**) Lung; (**b**) colon; (**c**) legs.

In this study, the execution time was independent of the distribution of density values such as bone and soft tissue arrangement, because we performed the same operation for each cell. However, the execution time was related to the transformation pattern and size of the volume data. Applying each transformation to various data, the execution time showed a proportional relationship with the size of the data. Table 4 presents the results of applying Wave transform to various data. The rendering results on the lung, colon, and legs data used are shown in Figure 8.

## 4. Conclusions

We proposed a novel efficient method of parallel resampling by developing volume-based parallel resampling. In modern deformation modeling, the number of cells increases significantly as the size of cells decreases in order to implement sophisticated movements. Thus, considering the fact that texture sampling and inverse matrix calculation are time consuming in existing methods, we highlighted the cause of the problems and suggested new methods to solve them.

In order to calculate the inverse transformation in the deformation simulation, the inverse matrix calculation using the vertex positions of the tetrahedron is frequently used. Considering that the error is related to the size of the elements of the matrices when calculating the inverse matrices, a method of maintaining smaller matrix elements was described. When the number of cells increased, the correct calculation could be performed solely by using the double in the existing method. Since modern GPUs are optimized for floats rather than doubles, this leads to performance degradation.

In this study, we set a reference point for each tetrahedron, and all vertices inside the tetrahedron were moved in parallel closer to the origin, as the reference point moved to the origin. The first advantage of the proposed method is that each element of the matrix becomes close to zero. It was possible to calculate a large number of cells without deterioration of the output data, even when using float. The second advantage is that the number of operations required for the inverse matrix can be reduced, because a 3 × 3 matrix can be used instead of a 4 × 4 matrix. As a result, a 10 to 20 time improvement was seen in the results of the experiments on a laptop and a desktop computer.

In addition, we proposed a method to efficiently perform texture sampling. In a previous study, to process one cell, texture sampling was performed at each vertex for a total of eight samplings. The output values were calculated by interpolating the sampled values. As the number of cells increases, the required texture sampling also increases proportionally, and performance degradation occurs. In this study, texture sampling was performed at the output voxel by interpolating the coordinates in the undeformed space. As a result, the execution speed was improved by reducing the number of texture samplings.

In the experiments in this study, three different deformation patterns were proposed, and their performances were observed accordingly. We observed a performance improvement for all deformations when the deformation pattern was relatively simple. Therefore, the proposed approach is suitable for medical simulations where deformation occurs in a part of the human body. As a result of measuring the speed on a desktop and a low-end computer, such as a laptop, it was possible to realize an interactive speed of 10 fps. A real-time speed of 40 fps or more was maintained on a general desktop.

In this study, the speed was improved without any deterioration in image quality through the two proposed methods, and real-time deformable volume visualization was possible for volume data of a size used clinically. Applying a predefined formula to the volume transformation was a limitation of this study, but the proposed method is highly scalable, because another transformation method can be combined with the proposed method. Through this study, it is expected that the latency of medical imaging systems for massive data in the IoE environment will be improved.

## Figures and Tables

**Table 1:** Experimental CT image size.

	Size	Capacity
Abdomen	512 × 512 × 300	150 MB
Lung	512 × 512 × 316	158 MB
Colon	512 × 512 × 141	70.5 MB
Leg	512 × 512 × 600	300 MB

**Table 2:** Resampling measurement results according to the inverse matrix calculation method.

	Desktop				Notebook			
Transform	No. Optimizations (a)	3D Double (b) [19]	Our 3D Float (c)	(a)/(c)	No. Optimizations (a)	3D Double (b) [19]	Our 3D Float (c)	(a)/(c)
Wave	348.14	150.55	22.55	15.43x	892.85	360.26	60.40	14.78x
Twist	376.11	166.57	26.56	14.16x	989.42	415.30	98.55	10.03x
Bubble	385.36	168.49	17.44	22.09x	940.06	402.53	58.39	16.09x

**Table 3:** Resampling measurement results according to the interpolation method.

	Desktop			Notebook		
Transform	Aguilera’s Method [16] (a)	Proposed Method (b)	(a)/(b)	Aguilera’s Method [16] (a)	Proposed Method (b)	(a)/(b)
Wave	22.55	19.75	1.14x	60.40	51.27	1.17x
Twist	26.56	23.82	1.11x	98.55	90.78	1.09x
Bubble	17.44	14.08	1.23x	58.39	47.18	1.23x

**Table 4:** Resampling of measurement results by interpolation on multiple medical data (Wave transform).

Data	Aguilera’s Method [16]	Proposed Method
Abdomen (300)	60.40	51.27
Lung (316)	63.53	54.87
Colon (141)	29.37	24.46
Legs (600)	149.37	127.19

## Data Availability

Data available on request due to restrictions eg privacy or ethical.

## Appendix A

template <typename T> /* T can be float or double */ T Determinant ( ) { T n11 = 255.9, n12 = 256.7, n13 = 256.7, n14 = 255.9; T n21 = 256.7, n22 = 255.9, n23 = 256.7, n24 = 255.9; T n31 = 133.1, n32 = 133.4, n33 = 132.3, n34 = 132.3; T n41 = 1, n42 = 1, n43 = 1, n44 = 1; T t11 = n23*n34*n42 − n24*n33*n42 + n24*n32*n43 − n22*n34*n43 − n23*n32*n44 + n22*n33*n44; T t12 = n14*n33*n42 − n13*n34*n42 − n14*n32*n43 + n12*n34*n43 + n13*n32*n44 − n12*n33*n44; T t13 = n13*n24*n42 − n14*n23*n42 + n14*n22*n43 − n12*n24*n43 − n13*n22*n44 + n12*n23*n44; T t14 = n14*n23*n32 − n13*n24*n32 − n14*n22*n33 + n12*n24*n33 + n13*n22*n34 − n12*n23*n34; T det = n11*t11 + n21*t12 + n31*t13 + n41*t14; return det; }

## References

[1] Nienhuys H.W., Frank van der Stappen A. A surgery simulation supporting cuts and finite element deformation. Proceedings of the Fourth International Conference on Medical Image Computing & Computer-Assisted Intervention.

[2] Heng P.A., Cheng C.Y., Wong T.T., Xu Y., Chui Y.P., Chan K.M., Tso S.K. (2004). A virtual-reality training system for knee arthroscopic surgery. IEEE Trans. Inf. Technol. Biomed..

[3] Si W., Lu J., Liao X., Wang Q. (2018). Towards interactive progressive cutting of deformable bodies via phyxel-associated surface mesh approach for virtual surgery. IEEE Access.

[4] Gibson S.F. 3D Chainmail: A Fast Algorithm for Deforming Volumetric Objects. Proceedings of the ACM Siggraph Symp Interact 3D Graph Games 1997.

[5] Liu T., Bargteil A.W., O’Brien J.F., Kavan L. (2013). Fast simulation of mass-spring systems. ACM Trans. Graph (TOG).

[6] Berndt I., Torchelsen R., Maciel A. (2017). Efficient surgical cutting with position-based dynamics. IEEE Comput. Graph. Appl..

[7] Rößler F., Wolff T., Ertl T. Direct GPU-based Volume Deformation. Proceedings of the CURAC.

[8] Kwon K., Chae S., Shin B.S. (2011). Anti-aliasing on deformed area using adaptive super sampling during volume ray-casting. Biomed. Eng. Lett..

[9] Rezk-Salama C., Scheuering M., Soza G., Greiner G. Fast volumetric deformation on general purpose hardware. Proceedings of the ACM SIGGRAPH/EUROGRAPHICS Workshop on Graphics Hardware.

[10] Correa C.D., Silver D., Chen M. Discontinuous displacement mapping for volume graphics. Proceedings of the VG@ SIGGRAPH.

[11] Herrera I., Buchart C., Aguinaga I., Borro D. (2014). Study of a ray casting technique for the visualization of deformable volumes. IEEE Trans. Vis. Comput. Graph..

[12] Schulze F., Bühler K., Hadwiger M. Interactive deformation and visualization of large volume datasets. Proceedings of the GRAPP (AS/IE).

[13] Murray L.M., Lee A., Jacob P.E. (2016). Parallel resampling in the particle filter. J. Comput. Graph. Stat..

[14] Nicely M.A., Wells B.E. (2019). Improved parallel resampling methods for particle filtering. IEEE Access.

[15] Gascon J., Espadero J.M., Perez A.G., Torres R., Otaduy M.A. Fast deformation of volume data using tetrahedral mesh rasterization. Proceedings of the 12th Computer Animation.

[16] Aguilera A.R., Salas A.L., Perandrés D.M., Otaduy M.A. (2015). A parallel resampling method for interactive deformation of volumetric models. Comput. Graphs.

[17] Torres R., Rodríguez A., Otaduy M. (2021). Hands-On Deformation of Volumetric Anatomical Images on a Touch screen. Appl. Sci..

[18] Chen J., Tai K.W., Chen W.C., Ouhyoung M. (2021). Robust Voxelization and Visualization by Improved Tetrahedral Mesh Generation. arXiv.

[19] Teschner M., Heidelberger B., Müller M., Pomerantes D., Gross M.H. (2003). Optimized spatial hashing for collision detection of deformable objects. Proc. Int. Fall Workshop Vis. Model Vis..

[20] Levoy M. (1988). Display of surfaces from volume data. IEEE Comput. Graph. Appl..

[21] Kim J., Ha T., Kye H. (2019). Real-Time Computed Tomography Volume Visualization with Ambient Occlusion of Hand-Drawn Transfer Function Using Local Vicinity Statistic. Healthc. Inform. Res..

